# *Pasteurella Multocida* meningoencephalomyelitis in a dog secondary to severe periodontal disease

**DOI:** 10.1002/ccr3.1561

**Published:** 2018-04-26

**Authors:** Alexander E. Tun, Leontine Benedicenti, Evelyn M. Galban

**Affiliations:** ^1^ School of Veterinary Medicine Matthew J. Ryan Veterinary Hospital University of Pennsylvania Philadelphia PA USA

**Keywords:** blood–brain barrier, meningoencephalomyelitis, *Pasteurella multocida*

## Abstract

*Pasteurella multocida* can cause meningoencephalomyelitis in canine patients with severe periodontal disease. Fever and neutrophilic pleocytosis in the cerebrospinal fluid are likely, and blood culture and/or empiric antibiotic therapy are indicated.

## Case presentation

A 5‐year‐old 5.14 kg (11.3‐lb) spayed female Cavalier King Charles Spaniel was presented to the Neurology and Neurosurgery service at the Matthew J. Ryan Veterinary Hospital of the University of Pennsylvania for evaluation of ataxia and cervical hyperesthesia. The day prior the patient was initially presented to the referring veterinarian for a 2‐day history of lethargy and inappetence. Physical examination revealed a rectal temperature of 40.2°C (104.4°F) and severe periodontal disease and was otherwise unremarkable. A similar episode occurred several months earlier and was suspected to be due to the periodontal disease. A dental cleaning had been recommended at the time but not pursued and the signs resolved with a 2 week course of both oral metronidazole (9.7 mg/kg [4.4 mg/lb], PO, q 12 h) and amoxicillin trihydrate, clavulanate potassium^1^ (12.1 mg/kg [5.5 mg/lb], PO, q12 h). The patient was hospitalized overnight on intravenous fluids and started on ampicillin sodium/sulbactam sodium (30 mg/kg [13.6 mg/lb], IV, q 8 h) based on previous response to antibiotic therapy. The following day the dog was referred for evaluation by the Dentistry and Oral Surgery service.

Physical examination at the time of referral revealed a rectal temperature of 39.4°C (102.9°F), a heart rate of 160, and a respiratory rate of 48 with shallow breaths. Occasional premature heartbeats were noted on auscultation. Mucous membranes were dark pink with a capillary refill time of approximately 1 sec. Moderate dental calculus was noted. The patient had also developed an unclassified ataxia and cervical hyperesthesia.

Consultation by the Dentistry and Oral Surgery Service confirmed the severe periodontal disease with contact ulceration and possible necrotizing stomatitis. However, the changes were not considered to be to the degree that would cause lethargy and inappetence, and so dental cleaning with extractions was recommended once the patient condition had stabilized.

Neurologic consultation identified that the dog had a dull mentation. On cranial nerve examination, an inconsistent bilateral menace response was noted. A low head and neck carriage, with a wide‐based stance in all four limbs, was also noted. The dog was ambulatory tetraparetic, tending to knuckle the thoracic limbs and listing and falling to either side suggesting a component of vestibular ataxia in addition to the more obvious general proprioceptive ataxia. The dog had postural reaction deficits noted during paw placing (absent) and hopping (delayed to absent) in all four limbs. The segmental reflexes in both pelvic limbs were increased, more so on the right, with increased muscle tone in that limb. The dog had moderate cervical hyperesthesia on palpation and flexion. Neuroanatomical diagnosis was consistent with a multifocal process with both intracranial and cervical myelopathy signs. Differential diagnoses included an infectious or inflammatory MEM, with neoplasia considered less likely.

Complete blood count revealed a mild normocytic normochromic anemia (HCT 39.2%; reference range 40.3–60.3%), a mild thrombocytopenia (127 × 10^3/*μ*L; reference range 177–398 × 10^3/*μ*L), and a normal total white cell count although few mild toxic change cells were noted and band cells were present (274/*μ*L). Serum biochemical profile revealed a slight hypokalemia (3.9 mmol/L; reference range 4.0–5.2 mmol/L) and a moderately elevated alkaline phosphatase (472 U/L; reference range 20–155 U/L). A 4DX SNAP^2^ test was negative. Urinalysis was unremarkable, and urine culture was negative. Abdominal ultrasound revealed no sonographic changes to explain the fever.

The patient had an AA stabilization 4 years prior via a ventral approach with seven positive profile pins and polymethyl methacrylate. Given the neurolocalization and the cervical implants, a CT precontrast and postcontrast of the head and neck was performed using a 16‐slice MDCT unit^3^ instead of MRI. The left transarticular pin was found to be broken within the atlas with the distal end displaced roughly 1.7 mm laterally although the radiologist commented it had not migrated significantly. The pin directed at the right pedicle of the atlas as well as the tip of the right transarticular pin breached the spinal canal. The metal bloom and streak artifact from the implants prevented any interpretation of spinal cord impingement. It was possible that these pins were in contact with or penetrating the meninges along the right lateral surface of the spinal cord. There was no clear evidence of osteomyelitis around the implants. There was normal alignment of C1 and C2. On the postcontrast images, there was mild contrast enhancement of the epidural/meningeal tissues immediately cranial to the implants at the level of the atlanto‐occipital junction. Several additional abnormalities were noted in the brain and cervical spine: congenital vertebral malformations at C6‐C7 (C7 spina bifida, malformed right dorsal lamina of C6 and C7, and abnormal articular process joints), mild third and fourth ventricle dilation, and supracollicular fluid accumulation. The left tympanic bulla was filled with a soft tissue density consistent with either primary secretory otitis media or otitis media. There was mild enlargement of the mandibular and medial retropharyngeal lymph nodes.

Following CT, CSF was collected from the fifth and sixth lumbar (L5 to L6) intervertebral site. Cerebrospinal fluid analysis revealed nucleated cell count 42/*μ*L, red blood cell count 310/*μ*L, and total protein 248 mg/dL, with 83% nondegenerative neutrophils, 13% large mononuclear cells, and 4% small lymphocytes. No microorganisms or neoplastic cells were identified.

Blood was drawn from both saphenous veins for aerobic culture. The dog was then started on empiric therapy for bacterial meningitis with ceftazidime^4^ (40 mg/kg [18.1 mg/lb], IV, q 6 h) and clindamycin^5^ (10 mg/kg [4.5 mg/lb], IV, q 12 h) for protozoal coverage. The dog was maintained on anti‐emetics and gastroprotectants, an opioid for analgesia, and dexamethasone sodium phosphate^6^ (0.1 mg/kg [0.045 mg/lb], IV, q 12 h) as an anti‐inflammatory.

The following day the dog's mentation, ataxia, and appetite mildly improved. Within 2 days, the dog was eating well and was transitioned to oral medications. Preliminary blood and CSF culture results suggested *Pasteurella* sp. growth. Pending final culture results oral clindamycin HCL^7^ was continued (9.7 mg/kg [4.4 mg/lb], PO, q 12 h) and enrofloxacin^8^ (13.2 mg/kg [6 mg/lb], PO, q 24 h) was initiated. The dog was also continued on a short tapering course of oral prednisone^9^ for 5 days (0.5 mg/kg/day to 0.25 mg/kg/day [0.22 to 0.11 mg/lb/day]) and gabapentin^10^ was used for analgesia (5 mg/kg [2.27 mg/lb], PO, q 8–12 h). After several more days of monitoring, the dog was discharged home.

*Pasteurella multocida* was isolated from blood (right saphenous vein only) and CSF cultures several days later. In addition, *Staphylococcus epidermidis* was isolated from CSF culture, although this was suspected to have been a contaminant. The *P. multocida* sensitivity panel matched on the isolates from blood and CSF, with susceptibility to all antibiotics tested except clindamycin to which it was resistant. As the *P. multocida* was susceptible to enrofloxacin, the prescribed antibiotic regimen was continued until recheck.

The dog was returned for evaluation 8 days after discharge. The dog was reported to be doing well, eating consistently, and walking significantly better. An occasional yelp was reported, but the dog seemed comfortable overall. Physical examination was unremarkable aside from unchanged periodontal disease. Neurologic examination revealed normal mentation and menace response, a mild wide stance in the pelvic limbs with no ataxia, mild postural reaction deficits in the left thoracic limb, mildly increased pelvic limb stretch reflexes, and mild cervical discomfort on palpation consistent with a cervical myelopathy. Clindamycin was discontinued after completing a 2‐week course, while enrofloxacin was continued for a total of 6 weeks. Reexamination after 4 weeks with repeat CSF analysis and culture and dental cleaning with extractions was planned with the owner, but the patient did not return for follow‐up. Fifteen months later, the patient was presented to the Dentistry and Oral Surgery Service for evaluation about a professional dental cleaning and extractions and was reported to be neurologically normal.

## Discussion

*Pasteurella multocida* is a small gram‐negative coccobacillus. It is a facultative anaerobe that is part of the normal flora of the respiratory tract and oral cavity of many mammals. Carriage rates are reported to be highest among cats (70–90%) and dogs (20–50%) 1, 2. *Pasteurella multocida* meningitis is a rare clinical occurrence in humans and in veterinary medicine 1. To date, it has only been reported in the veterinary literature in two cats, both with bite wounds, and a dog with an ocular infection that spread to the brain 3, 4. Human and veterinary patients typically present with similar signs, including fever, headache, nuchal rigidity, nausea, and altered level of consciousness 1. Cerebrospinal fluid analysis is consistent with a bacterial meningitis, with leukocytosis (typically >50% neutrophils), elevated protein level, and decreased glucose 1. Interestingly, up to 89% of human patients report some form of animal exposure, with nonbite exposures being more common, and bites only documented in 15% of cases 1.

Deposition of *P. multocida* on injured skin or mucosal surfaces by licking may be the most common form of human exposure, reported in up to 75% of cases 1. *Pasteurella multocida* is suspected to cause meningitis by direct inoculation by an animal bite penetrating the skull, contamination from colonized sites by skull fracture or cranial surgery, local spread from an adjacent site, or hematogenous seeding from bacteremia 1. Hematogenous seeding is the most common cause of *P. multocida* meningitis in humans, while only direct inoculation, injury, or invasion has been reported in the veterinary literature 1. As the patient described had no such inoculation or injury, it is the hypothesis of the authors that this patient's severe periodontal disease leads to a bacteremia causing hematogenous seeding of a bacterial meningitis originating at the disrupted blood–spinal cord barrier. Several aspects of the patient history are supportive of this theory.

First, *P. multocida* is a common oral bacteria in dogs. The patient previously had similar clinical signs and findings of oral disease consistent with infection that resolved with the administration of metronidazole and amoxicillin trihydrate, clavulanate potassium. The *P. multocida* cultured in the patient was susceptible to the latter.

Second, a thorough search for source of bacterial infection was performed. There was soft tissue material in the left tympanic bulla on CT, although there was no evidence of bone lysis or proliferation or a brain stem lesion in the region of the tympanic bulla suggestive of an ascending infection. In addition, *P. multocida* is not commonly isolated from the middle ear in canine patients with otitis media/interna, although it is in people 1, 5. Ideally, this material might have been sampled to confirm primary secretory otitis media. The CSF culture also grew *S. epidermidis*, a common skin contaminant from humans. The susceptibility profile was different from that of the *P. multocida*, and it was elected to not expand antibiotic coverage to address that organism. As the patient improved in the face of this decision, this would further support our interpretation, emphasizing the importance of interpreting culture results carefully. No other source of infection was found.

Finally, the ventral system of venous sinuses (ventral cerebral veins and ventral petrosal and cavernous sinuses) drains the ventral forebrain, as well as the face, nasal cavity, orbit, and upper teeth. As a result, infective material from those areas can drain directly into this system of sinuses and spread to the neuraxis 6.

It is interesting to consider the frequency of severe periodontal disease in dogs and the rarity of documented spread to the central nervous system. We considered the specific reasons for this patient that allowed for spread of infection. In humans, the transition of oral commensal bacteria to pathogens is still not well understood, but may involve the complexity (ratio of gram‐positive to gram‐negative bacteria) and volume of the microbiome, and changes in the host immune response 7. The patient did not have any underlying cause for immunosuppression that might have increased susceptibility.

It is unclear what if any significance the dog's surgical implants may have had in this case. As they were present for 4 years prior to this issue and there was no evidence of osteomyelitis on CT, a chronic biofilm was considered less likely. It was entertained that they might have provided a surface for colonization or caused meningeal irritation or penetration allowing the blood–spinal cord barrier to be breached and infection to spread. Postoperative radiographs were performed at the time of AA stabilization, making comparison with the cross‐sectional imaging difficult. However, there was no evidence that the pins migrated or that there was any change in the stability of the apparatus over time, despite one of the pins breaking. It is also difficult to assess from imaging if the pins had contact with or penetrated the meninges. Given their position in the canal, either of these conditions is plausible.

It is possible that similar infections occur and go undetected. Patients presenting with fever of unknown origin often receive empiric antibiotic therapy. The choices for this therapy align with the relative susceptibility of *P. multocida*. It is possible that CNS infections are being regularly treated without definitive diagnosis. The antibiotics selected empirically prior to culture results were meant to provide broad‐spectrum coverage of the CNS. Transitioning to oral antibiotics with knowledge that the bacteria were gram‐negative, enrofloxacin was selected. In humans with *P. multocida* meningitis, empiric coverage typically starts with penicillins or third‐generation cephalosporins 1, 8, 9. Chloramphenicol has been recommended if a beta‐lactam antibiotic allergy exists, although this is no longer recommended in veterinary patients due to concern about risk of relapse as the drug is bacteriostatic 10. The optimal duration of therapy is not well known in veterinary patients or humans, although in humans 10–14 days is considered appropriate for most patients pending clinical response 1, 10.

In those patients where a bacterial MEM is suspected, the cause may not be confirmed as the culture rate for CSF in canines is low, reportedly between 13% and 39% 11. Positive blood cultures have been reported in up to 63% of human patients with *P. multocida* meningitis 1. In a case series of 23 dogs with bacterial MEM, only three had blood cultures, one of which was positive, so no significant conclusions can yet be made about the likelihood of identifying a positive blood culture in canine patients with bacterial CNS infection 11. The patient described here was positive in only one of the two blood samples cultured, again reflecting the challenge of successfully obtaining a positive blood culture for confirmation.

## Concluding remarks

The patient described exhibited clinical signs and laboratory findings consistent with previous reports of bacterial MEM in dogs. Based on CSF and blood culture, as well as response to therapy, a diagnosis of *P. multocida* meningoencephalomyelitis was made. This is the first report of a canine patient with a *P. multocida* CNS infection without evidence of a direct inoculation. In addition, this is the first report where the source of the infection is suspected to be severe periodontal disease. Further support for this theory might have come from concurrent culture of the oral flora or any extracted teeth with matching susceptibility pattern. In the future, be aware that a fever with multifocal neurologic signs and severe periodontal disease in a canine patient may suggest a *P. multocida* infection and both CSF and blood cultures can be submitted for confirmation.

## Authorship

AT: analyzed and interpreted the patient data, designed and wrote the manuscript. LB: helped analyze and interpret the patient data, and contributed to writing the manuscript. EG: contributed to writing the manuscript. All authors: read and approved the final manuscript.

## Conflict of interest

None declared.

## Notes

1 Clavamox (amoxicillin trihydrate, clavulanate potassium); Zoetis Inc, Kalamazoo, MI, USA

2 4DX SNAP; IDEXX Laboratories Inc, Westbrook, ME, USA.

3 GE BrightSpeed, General Electric Company, Milwaukee, WI, USA.

4 Ceftazidime; Sagent Pharmaceuticals, Schaumburg, IL, USA.

5 Clindamycin; Alvogen Inc, Pine Brook, NJ, USA.

6 Dexamethasone sodium phosphate; Fresenius Kabi USA LLC, Lake Zurich, IL, USA.

7 Clindamycin HCL; Wedgwood Pharmacy, Swedesboro, NJ, USA.

8 Baytril (enrofloxacin); Bayer HealthCare LLC, Animal Health Division, Shawnee Mission, Kansas.

9 Prednisone; West‐Ward Pharmaceutical Corp, Eatontown, NJ, USA.

10 Gabapentin; Letco Medical LLC, Decatur, AL, USA
